# Editorial: Neurodevelopmental and behavioral outcomes following a traumatic event in infancy

**DOI:** 10.3389/fpsyg.2023.1157991

**Published:** 2023-04-26

**Authors:** Vito Giordano, Philipp Deindl, Renate Fuiko, Oswald Kothgassner

**Affiliations:** ^1^Division of Neonatology, Pediatric Intensive Care and Neuropediatrics, Comprehensive Center for Pediatrics, Medical University of Vienna, Vienna, Austria; ^2^Department of Neonatology and Pediatric Intensive Care Medicine, University Children's Hospital, University Medical Center Hamburg-Eppendorf, Hamburg, Germany; ^3^Department of Child and Adolescent Psychiatry, Comprehensive Center for Pediatrics, Medical University of Vienna, Vienna, Austria

**Keywords:** neurodevelopment, spinal cord injury, COVID-19, mother–infant relationships, bully behavior

This Research Topic presents new insights into the impact of traumatic events on children's neurodevelopment and behavioral outcomes and includes two case reports and two original articles.

The neurodevelopment of a child is linked to their growth, maturation, and environment, making it crucial for outcome research to consider biological, cognitive, social, and emotional factors. The 1^st^ years of a child's life are especially important for their psychophysical formation, and sensitive phases exist that are affected by various elements such as age, sex, genetics, morpho-functional characteristics, social, and family environmental factors (Bowlby et al., 1989; Holmes, 2017).

Growing up in a healthy environment and experiencing the right sensory stimuli and social interactions are vital for a child's cognitive-behavioral development and their ability to handle future events (Rutter, 1980; Holmes, 2017).

Critical events at various developmental stages can affect a child's outcome, and global events' sudden cultural changes must be addressed (Klaver and Rohlfing, 2022).

The first case report, by Leon Machado et al., describes the physical and physiological consequences of spinal cord injury (SCI) and the impact it has on a child's development. They present a new neurotherapeutic approach based on activity-based restorative therapies (ABRT), including activity-based locomotor training (ABLT) (Harkema et al., 2010), and neuromuscular electrical stimulation (NMES) (Collins, 2007). This significantly improved trunk control, arm function, endurance, and alignment activation in a child with SCI in 59 ABRT sessions over 3 months. These improvements expanded the child's ability to engage in play and positively impacted family dynamics, as reported by the parents.

The second case report by Goode-Roberts et al. ABLT shows promising results for the treatment of premature male infants with incomplete cervical SCI using the ABLT approach. The child was treated 5 days per week for 9 months. Development was assessed for two different treatment periods using the Bayley Scale for Infant Development–Third edition (BSID-III). The authors describe improved trunk control, a sit-to-stand progression, a self-mobility progression, as well as improvement in hand motoric. In addition, looking at the BSID-III, an impressive improvement (still under the norm) was detected for scores in fine motoric (pre-intervention = 55; post intervention = 75).

Specialized neurorehabilitation approaches in SCI have been shown to have a positive impact on the child and families and should be a mandatory treatment for children with this relative rare but severe disease.

Just as an injury can directly impact the trajectory of child's development, an environment that is not congruent with a child's needs can have profound impact on children outcomes. The mother or caregiver plays a crucial role in a child's self-regulation processes, and parental mental health problems can significantly affect a child's psychophysical development and wellbeing (Ainsworth et al., 1974). A parental mental health problem can thus have significant consequences for the child's psychophysical development and wellbeing. The COVID-19 pandemic became a traumatic event for many people. It provoked fear of infection, death, restriction, limited access to health facilities, or isolation from family members, which also affected the parent-child dyad.

The authors Perez et al. investigated the impact of the pandemic on the mother-child dyad. The study found that compared to the control cohort, more mothers in the COVID-19 cohort reported depressive symptom levels above the cut-off values indicative of mild depressive disorders. Additionally, mothers in the COVID-19 group and those with higher depressive symptoms reported more infant regulatory problems regarding crying and sleeping.

Lastly, cyberbullying is a growing problem, among children and adolescents and can have severe traumatic consequences, sometimes resulting in extreme actions such as suicide (Peebles, 2014). Dantchev and Zemp provide valuable insights into the prevalence and impact of peer, sibling, and cyberbullying. They found that 24% of participants aged 9–15 reported frequent bullying, with cyber victimization occurring more often, while sibling victimization was more likely to be permanent. No significant gender differences were found in the context of bullying. Those who experienced victimization in one context were more likely to be victimized in another, and cyber victimization was associated with more conduct problems and higher academic achievement.

Overall, this Research Topic covers a range of medical and psychosocial conditions that can have potential traumatic effects on children across different contexts and age groups. The case reports demonstrate the efficacy of a new integrative neurotherapeutic approach, while the original articles highlight the critical role of mothers in infant regulation, the impact of the COVID-19 pandemic on parent-child dyads, and the prevalence and impact of peer, sibling, and cyberbullying.

## Author contributions

VG, PD, RF, and OK conceptualized and coordinated this Research Topic. All authors contributed to the article and approved the submitted version.
